# Stress-induced impairment reveals the stage and features of post-error adaptive adjustment

**DOI:** 10.3389/fnbeh.2022.1013170

**Published:** 2022-11-10

**Authors:** Na Hu, Quanshan Long, Dawei Zhang, Xiaoxi Wang, Min Deng, Qing Li, Minmin Yan, Antao Chen

**Affiliations:** ^1^School of Preschool and Special Education, Kunming University, Kunming, China; ^2^Faculty of Education, Yunnan Normal University, Kunming, China; ^3^School of Humanities and Management, Kunming Medical University, Kunming, China; ^4^Key Laboratory of Cognition and Personality of Ministry of Education, Faculty of Psychology, Southwest University, Chongqing, China; ^5^School of Psychology, Shanghai University of Sport, Shanghai, China

**Keywords:** acute stress, post-error adjustment, Trier Social Stress Test, response stimulus interval, cognitive control

## Abstract

An increased reaction time often occurs after error responses (post-error slowing, PES). However, the role of top-down regulation in post-error processing remains to be debated. Impairing cognitive control function through acute stress would help to investigate the role and stage of top-down adaptive regulation in post-error processing. Here, we recruited 50 healthy male participants who were randomly assigned to either a stress condition (Trier Social Stress Task, TSST) or a control condition (control version of the TSST). A color-word Stroop task with different response stimulus intervals (RSIs) was used to investigate the effects of acute stress on different stages of post-error processing. The results showed that cortisol, heart rate, perceived stress level, and negative affect were higher in the stress group (*n* = 24) than in the control group (*n* = 26), indicating successful stress induction. The accuracy of post-error response in the control group increased with the extension of RSI, and the reaction time decreased. However, the accuracy of 1,200 ms RSI was close to that of 700 ms RSI in the stress group but was significantly lower than that in the control group. The results suggested that acute stress caused the impairment of top-down adaptive regulation after error. Furthermore, our study manifested adaptive adjustment only in the late stages of post-error processing, indicating the phasic and adaptive features of post-error adjustment.

## Introduction

Error-induced adaptive adjustment is one of the critical functions of self-regulation in individuals, including two sub-processes of error monitoring and post-error adjustment (Gehring et al., 2018). Previous studies have shown that individuals slow down their reaction speed in the trials after making an error response in speeded response time tasks, a phenomenon termed post-error slowing (PES) (Laming, 1979). The conflict monitoring theory assumes that monitoring error responses would improve cognitive control for a better performance in post-error trials (Dutilh et al., 2012; Mattes et al., 2022). Brain imaging studies have found that neural signals from the anterior cingulate cortex (ACC) are transmitted to the dorsolateral prefrontal cortex (PFC) under error monitoring, which enhances cognitive control in subsequent responses (Yeung et al., 2004; Danielmeier et al., 2011). However, since some have studies found less accurate responses after error (Dudschig and Jentzsch, 2009), the cause and explanation of this effect remain to be debated (Danielmeier and Ullsperger, 2011). It has been proposed that PES is not driven by up-regulation in cognitive control but rather by impaired task-related processing in the subsequent trials caused by error monitoring. The orientation account suggests that the orienting response to error responses results in a slower post-error response (Notebaert et al., 2009; van den Brink et al., 2014). On the other hand, the error-monitoring hypothesis argues that the occupancy of cognitive resources from error monitoring in post-error processing causes the slowing down of response (Dudschig and Jentzsch, 2009).

Recent studies have further revealed different sub-stages in post-error processing (Steinhauser et al., 2017; Wessel and Aron, 2017; Guan and Wessel, 2021). Specifically, researchers used experimental tasks with different response stimulus intervals (RSIs) or stimulus onset asynchrony (SOA) to explore the processing characteristics in different stages of post-error adjustment. The results showed prolonged reaction time and reduced response accuracy in the early stage after the error responses. However, the reaction time after the error gradually shortened, and the accuracy increased even better than in post-correct responses during the late processing stage (Danielmeier et al., 2015; Van der Borght et al., 2016). The error monitoring caused interference in the early processing stage of post-error trials. Only in the late processing stage can individuals improve behavioral performance through adequate selective attention to task-related information. It indicated that maladaptive processing turns into adaptive regulation during post-error adjustment as time passes (Buzzell et al., 2017; Li et al., 2021). Above all, there exist multiple accounts for the role of top-down adaptive regulation in post-error response. In fact, post-error improvement in accuracy (PIA) can help confirm that post-error processing is adaptive or maladaptive in different processing stages. However, not all experiments revealed PES and PIA simultaneously (Hajcak and Simons, 2008; Beatty et al., 2018), which may depend on experimental tasks, task difficulty, error awareness, SOA/RSI, and so on. Therefore, we can explore the adaptive or non-adaptive characteristics of post-error processing by modulating the cognitive control function in individuals to avoid interference from potential influencing factors.

Stress is recognized as an essential factor in impairing high-order prefrontal cortex functions (Arnsten, 2009, 2015). When encountering acute stress, homeostasis of the body is rapidly disrupted. The stress response in the body is to regain balance in a threatening situation (Dickerson and Kemeny, 2004). The stress responses were mainly regulated by the activity of the sympathetic–adrenal–medullary (SAM-axis) and hypothalamic–pituitary–adrenal (HPA-axis) axes (Allen et al., 2014). Under stressful situations, the SAM axis is rapidly activated. It releases large amounts of catecholamines, leading to heart rate, blood pressure, and respiration changes, resulting in the body's “fight-or-flight” response. In addition, stress-level catecholamine and dopamine enhance the emotional response mediated by the amygdala and weaken the higher-order PFC functions (Arnsten, 2015). Furthermore, as a product of the HPA axis, cortisol is vital in mobilizing body resources in response to current stressors (Ulrich-Lai and Herman, 2009). Accumulating evidence suggests that the stress level of cortisol enhances the catecholaminergic effects in these regions through glucocorticoid or mineralocorticoid receptors (De Kloet et al., 2005; Roozendaal et al., 2006). The biphasic–reciprocal model proposed by Hermans et al. (2014) reveals that when exposed to stress, the stress levels of catecholamines increase the activation of the salience network while weakening the activity of the executive control network. In this way, stress enhances the emotional and vigilance responses of individuals but impairs high-order cognitive control function. In addition, evidence has revealed impaired core executive functions following acute stress (Qin et al., 2009; Plessow et al., 2011; Sänger et al., 2014). Accordingly, acute stress might reduce top-down attention regulation after error responses.

This study aims to reveal the processing characteristics of adaptive regulation in post-error adjustment. We can explore the role and stages of adaptive regulation in post-error processing by impairing the top-down cognitive control through acute stress. Based on previous studies, dividing post-error processing into different stages could help to explore adaptive or maladaptive processing characteristics in post-error adjustment. Here, we used the Trier Social Stress Test (TSST) to induce stress responses in the stress group and explored the stress effect on the multiple-stage post-error processing through the Stroop task, which contained three kinds of RSIs (200, 700, and 1,200 ms). The manipulation of RSIs was largely based on the study by Buzzell et al. (2017). In contrast, the participants in the control group completed the control-TSST. We measured the physical and psychological responses in the stress and control groups during the experiment by collecting the salivary cortisol, heart rate, perceived stress levels, and positive and negative emotions to determine whether the stress response was successfully induced. By comparing the post-error behavioral responses under different RSIs in the stress and control groups, we investigated the stress effects on multiple-stage processing of the post-error adjustment. In accordance with the post-error multi-stage processing theory, which supports post-error response including both adaptive and maladaptive processing stages, we expect longer RT and lower accuracy in the post-error trials than those in the post-correct trials at the 200 ms RSI for both groups. Moreover, the accuracy of the post-error trials would increase over time, and the amount of the post-error adjustment would be highest at the 1,200 ms RSI for the control group. There would be longer RT and lower accuracy in the post-error trials at the 700 and 1,200 ms RSIs for the stress group than for the control group.

## Methods

### Participants

Given the effects of gender differences and menstrual cycles on individuals's stress responses and cognitive processing (Kudielka and Kirschbaum, 2005; Laredo et al., 2015), only male participants (n = 55) were recruited for this experiment. This experiment recruited participants through Internet advertisements and further interviewed by telephone. All participants were healthy, right-handed, nonsmokers, and had no medication during the prior week. To exclude individuals with depression and chronic stress, participants filled out the online questionnaires, including the Life Events Scale (LES) (Zheng and Yang, 1990) and the Beck Depression Inventory (BDI) (Beck, 1976). Only individuals with an LES score under 20 and a BDI score under 8 (with no depressive symptoms) were recruited for this experiment. Five participants were eliminated due to error trials being deficient (fewer than six trials under a single RSI condition) or due to missing saliva samples. There were 24 male participants in the stress condition and 26 male participants in the control condition (*M* ± *SD*: 20.56 ± 1.39 years). Mean body mass index (BMI): 20.57 ± 2.23, Life Events Scale score: 9.68 ± 6.22, and Baker Depression Scale score: 3.32 ± 2.50. All participants signed informed consent and were uninformed about the experimental purpose before the experiment ended. The study was approved by South-west University Human Ethics Committee for Human Research.

### Experimental procedure

**Figure 1:**
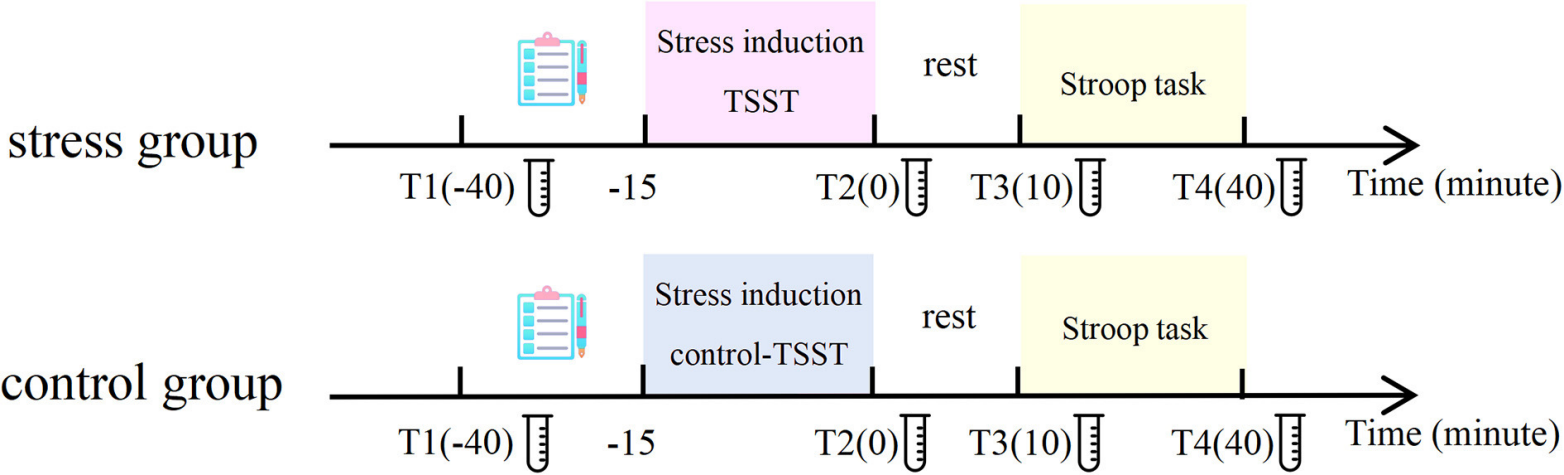
General view of the experimental procedure.

The experimental procedure is displayed in Figure 1 and it lasted for a duration of approximately 1.5 h. This experiment was performed between 1:00 p.m. and 7:00 p.m. owing to individuals' relatively low and stable endogenous cortisol levels in the afternoon. The participants were randomly exposed to the stress condition or the control condition. When participants arrived at the laboratory, they took a 10-min rest. Then, participants were fitted with the electrocardiogram (ECG) acquisition device and filled out the State-Trait Anxiety Inventory (STAI). Then, the first saliva cortisol samples and the first heart rate samples were collected. Next, participants filled out the Perceived Stress Assessment Scale and Positive and Negative Affect Schedule (PANAS) for the first time (T1). Participants rated the state of stress on a scale of 1–10 through the Perceived Stress Assessment Scale, with a higher score manifesting a higher stress level. Subsequently, the participants performed the practice blocks of the Stroop task. Then, the participants in the stress group performed the TSST, and the control group performed the control version of the TSST. Immediately after the stress/control induction, the second samples of salivary cortisol, heart rate, perceived stress level, and PANAS were collected from each participant (T2). After a 10-min rest waiting for the peak concentration of stress-level cortisol to be reached, we collected the third samples of salivary cortisol, heart rate, perceived stress level, and the PANAS (T3). The participants then completed the formal experimental task. Participants sat in a soundproof room 60 cm away from a 17-inch monitor (85 Hz refresh rate, 1,024 × 768 resolution). E-Prime software (E-Prime 2.0, Psychological Software Tools, Pittsburgh, PA, USA) controlled the experiment. After finishing, we collected the fourth samples of salivary cortisol, heart rate, perceived stress level, and PANAS from each participant (T4).

### Color-word Stroop task

This experiment used the adapted color-word Stroop task to explore the post-error processing. The task used four colors and four corresponding Chinese characters to each color as stimuli. The color consisted of red [RGB (red green blue):255,0,0], blue (0,0,255), green (0,255,0), and yellow (255,255,0). The stimuli types were color-word incongruent or color-word congruent. The meaning of the color word conflicted with the font color in the color-word incongruent trial (e.g., red written in the yellow font), while the meaning of the color word was the same as the font color in the color-word congruent trial (e.g., green written in green font). The Stroop stimulus subtended 1.83 degrees. The Stroop stimulus was presented on a black background. The participants were asked to use the index and middle fingers of both hands to respond to the color of the font as accurately and quickly as possible while ignoring the meaning of the words. The reaction keys were D, F, J, and K on a standard keyboard. Precisely, the four colors corresponded to the D (left middle finger), F (left index finger), J (right index finger), and K (right middle finger) keys. The key assignments were counterbalanced across participants. In each trial, the color word was presented in a white box in the center of the screen at most for 1,500 ms and terminated after pressing any response key. The interval between trials was 200, 700, and 1,200 ms. Since error consciousness modulates post-error regulation (no post-error regulation following unaware error responses) (Hester et al., 2005), the trial interval presented the task feedback to rule out the interference from error consciousness. When the response was correct, it presented “√”; but when it was wrong, it presented “×.” A “MISS” was presented when the participants did not press any response key before the deadline. Each participant must complete three practice blocks which contain 20 trials in each block before the experimental sessions. Only when accuracy in the practice block exceeded 85%, were participants allowed to perform the formal experiment. Five blocks with 960 trials with 480 congruent trials and 480 incongruent trials each were presented in the formal experiment. The numbers of congruent and incongruent trials were equal in the three RSIs. The total task duration is about 25 min.

### Stress induction and stress validation

The participants in the stress condition performed the TSST (Kirschbaum et al., 1993), while the control group performed the control version of the TSST. The task includes a 5-min preparation phase and a 10-min test phase. The participants were informed of the stress tasks at the preparation phase. The test phase consists of speech (5 min) and mental arithmetic (5 min) tasks. Specifically, participants were required to perform a job interview (e.g., teacher) and try their best to get the job in the speech task. In the mental arithmetic task, participants were informed to count backward from 2,043 in steps of 17 as accurately and quickly as possible. When an incorrect figure was recited, they had to start counting from 2,043. A camera videotaped the participants throughout the tasks. In addition, two experimenters monitored and evaluated the participants' performance without facial expressions. Finally, the research team informed participants that their performances were not so good and that they had to perform the TSST again. The participants in the control condition shared the same time course as the participants in the stress condition, but the tasks were more manageable. Specifically, participants talked about a novel, movie, or a recent vacation trip and counted forward from 0 in steps of 15 at their own pace without being videotaped or commissioned.

This experiment measured the participants' stress levels through salivary cortisol, heart rate, perceived stress levels, and positive and negative affect. The saliva samples of participants were collected by a particular saliva collector (salivette, Sarstedt), and then the samples were stored at −20°C until analyzed. The enzyme-linked immunosorbent assay (ELISA) was used to detect the cortisol concentration (Engvall and Perlmann, 1971). The heart rate recording of the participants was completed by a multi-channel physiological signal recorder (MP150, BIOPAC, Goleta, USA) with a sampling frequency of 500 Hz. The ECG electrodes were placed on the chest, and the ECG data were analyzed in AcqKnowledge software (AcqKnowledge 4.2, BIOPAC, Goleta, USA). The heart rate was recorded at the same time points as the salivary cortisol sampling, and each heart rate sample was collected over 3 min. In addition, heart rate sampling during the TSST/control-TSST was continuous. The heart rate sampling at the T3 time point lasted 2 min to ensure the formal experiment was completed during the cortisol spike.

### Data analysis

#### Stress manipulation

In this experiment, the stress effects on the salivary cortisol, heart rate, perceived stress levels, and positive and negative affect were analyzed by repeated-measures analysis of variance (ANOVA) with factors Time (T1 to T4 time points) × Group (stress group vs. control group). In addition, the impact of the group on BMI, BDI, and LES was examined by independent sample *t*-tests. We analyzed the group difference in age by the chi-square test.

#### Stroop task

In this experiment, repeated-measures ANOVA with the factors Congruency (congruent vs. incongruent) × RSI (200 vs. 700 vs. 1,200 ms) × Group (control group vs. stress group) was used to examine the stress effect on the response times (RTs) and accuracy at different RSIs during the Stroop task.

#### Errors and post-error responses

The RTs during the correct and error trials were explored by repeated-measures ANOVA with the factors Trial Type × Group. Repeated-measures ANOVA with the factors Trial Type (post-error trial vs. post-correct trial) × RSI (200 vs. 700 vs. 1,200 ms) × Group (control group vs. stress group) was used to examine the stress effect on the RTs and accuracy of post-error responses under different RSIs.

#### Time-on-task effects

Considering that there are many trials in this task, we explored the fatigue effect in this experiment. The difference in accuracy and RTs between the first 27% and the last 27% of all trials during each participant was analyzed by the paired sample *t*-test. We also calculated the Stroop effect on the accuracy and RTs of all participants. In the same way, the difference in the Stroop effect between the first 27% and the last 27% of all trials was analyzed by the paired sample *t*-test.

The threshold of significance (α) for all statistical analyses was set to 0.05 (two-tailed). The alpha levels were corrected by the Bonferroni correction. Greenhouse–Geisser corrections were applied for sphericity. The least significant difference (LSD) test was applied when interaction effects were significant (Williams and Abdi, 2010). The partial eta squared (${\text{η}}_{\text{p}}^{2}$) was analyzed to indicate the effect sizes of the significant results.

With a sample size of 50 participants in this study, at a significance level of 0.05, and a population correlation of 0.60 in the repeated measures. The three-way interaction effect of Trial Type × RSI × Group on Accuracy and RTs can detect a medium effect (${\text{η}}_{\text{p}}^{2}$ = 0.25) at a probability (1–β) = 0.99. The two-way interaction effect of RSI × Group on Accuracy and RTs can detect a medium effect (${\text{η}}_{\text{p}}^{2}$ = 0.25) at a probability (1 – β) = 0.98.

## Results

### Stress data

#### Cortisol

**Figure 2:**
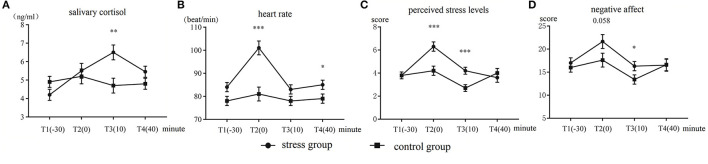
Mean and standard errors of **(A)** salivary cortisol levels, **(B)** heart rate, **(C)** perceived stress levels, and **(D)** negative affect in the stress group and control group. *p < 0.05, **p < 0.01, and ***p < 0.001.

The results of salivary cortisol are displayed in Figure 2A. The Time (T1–T4) × Group (control group vs. stress group) ANOVA of the cortisol levels showed a significant main effect of Time, *F*_(3,144)_ = 9.23, *p* < 0.001, ${\text{η}}_{\text{p}}^{2}$ = 0.16. The main effect of Group was not significant (*p* = 0.22). The Time × Group interaction was significant, *F*_(3,144)_ = 11.37, *p* < 0.001, ${\text{η}}_{\text{p}}^{2}$ = 0.19. Further analysis yielded that the main effect of Time was significant only within the stress group, *F*_(3,46)_ = 17.45, *p* < 0.001, ${\text{η}}_{\text{p}}^{2}$ = 0.52. The stress group had significantly higher cortisol levels than the control group at T3, *F*_(1,48)_ = 10.32, *p* = 0.002, ${\text{η}}_{\text{p}}^{2}$ = 0.17. No significant difference between the groups was observed at the other time points (*p*s > 0.08).

#### Heart rate

The heart rate results are displayed in Figure 2B. The Time × Group ANOVA disclosed a significant main effect of Time, *F*_(3,144)_ = 52.84, *p* < 0.001, ${\text{η}}_{\text{p}}^{2}$ = 0.51. The main effect of Group was significant, *F*_(1,50)_ = 8.67, *p* = 0.005, ${\text{η}}_{\text{p}}^{2}$ = 0.15. The interaction of Time × Group was also significant, *F*_(3,144)_ = 27.71, *p* < 0.001, ${\text{η}}_{\text{p}}^{2}=$ 0.36. The simple effect tests showed that the main effect of Time was only significant in the stress group, *F*_(3,46)_ = 43.99, *p* < 0.001, ${\text{η}}_{\text{p}}^{2}$ = 0.73. The heart rate of the stress group was significantly faster than that of the control group at T2, *F*_(1,48)_ = 26.29, *p* < 0.001, ${\text{η}}_{\text{p}}^{2}$ = 0.35 and T4, *F*_(1,48)_ = 3.77, *p* = 0.040, ${\text{η}}_{\text{p}}^{2}$ = 0.058. No significant difference between the groups was observed at the other time points (*p*s > 0.08).

#### Perceived stress level

The perceived stress-level results are illustrated in Figure 2C. The analysis results showed that the main effect of Time was significant, *F*_(3,144)_ = 25.63, *p* < 0.001, ${\text{η}}_{\text{p}}^{2}$ = 0.34. The main effect of Group was significant, *F*_(1,48)_ = 4.31, *p* = 0.043, ${\text{η}}_{\text{p}}^{2}$ = 0.08. The Group × Time interaction was also significant, *F*_(3,144)_ = 14.16, *p* < 0.001, ${\text{η}}_{\text{p}}^{2}$ = 0.22. Further analysis showed that the stress group had significantly higher perceived stress levels than the control group at T2, *F*_(1,48)_ = 15.56, *p* < 0.001, ${\text{η}}_{\text{p}}^{2}$ = 0.237, and T3, *F*_(1,48)_ = 12.39, *p* = 0.001, ${\text{η}}_{\text{p}}^{2}$ = 0.20. No significant difference between the groups was observed at the other time points (*p*s > 0.40).

#### Positive and negative affect

The negative affect results are illustrated in Figure 2D. The Group × Time ANOVA revealed a significant main effect of Time, *F*_(3,144)_ = 12.23, *p* < 0.001, ${\text{η}}_{\text{p}}^{2}$ = 0.20. The main effect of Group was not significant (*p* = 0.18). The interaction of Group × Time was significant, *F*_(3,144)_ = 2.80, p = 0.042, ${\text{η}}_{\text{p}}^{2}$ = 0.05. The simple effect tests yielded marginally significant differences at the T2 time point, *F*_(1,48)_ = 3.75, *p* = 0.058, ${\text{η}}_{\text{p}}^{2}$ = 0.07, and T3, *F*_(1,48)_ = 4.40, *p* = 0.04, ${\text{η}}_{\text{p}}^{2}$ = 0.08. Across the groups, the stress group had significantly higher perceived stress levels than the control group. No more significant difference between the groups was observed at the other time points (*p*s > 0.61).

Regarding the positive affect, the main effect of Time was significant, *F*_(3,144)_ = 324.49, *p* < 0.001, ${\text{η}}_{\text{p}}^{2}$ = 0.33, which showed that the scores of positive affect of the participants gradually decreased (M ± SD): T1 (27.75 ± 0.86) > T2 (26.38 ± 1.18) > T3 (24.79 ± 1.45) > T4 (21.96 ± 1.15). The main effect of Group and the interaction of Group × Time were not significant (*p*s > 0.17).

#### Other self-reported results

Independent samples *t*-tests for BMI, BDI, and LES showed no significant between-group difference (*p*s > 0.32), either for the chi-square test for age (*p* = 0.39). In addition, an independent samples *t*-test analysis of state and trait anxiety showed no significant between-group difference (*p*s > 0.24) (see Table 1).

**Table 1:** Characteristics of the participants in the stress and control groups (M ± SD).

	**Stress group**	**Control group**
Age	20.38 ± 1.21	20.73 ± 1.54
BMI	20.37 ± 1.85	21.50 ± 2.45
LES	9.19 ± 8.54	10.73 ± 6.17
BDI	3.08 ± 2.65	3.54 ± 2.37
Trait-anxiety	40.13 ± 9.08	43.19 ± 9.67

### Behavioral results

#### Stroop task

The accuracy and RTs of congruent and incongruent trials during different RSIs are illustrated in Table 2. The Congruency × RSI × Group ANOVA of the accuracy during the Stroop task showed a significant main effect of Congruency, *F*_(1,48)_ = 68.25, *p* < 0.001, ${\text{η}}_{\text{p}}^{2}$ = 0.59, indicating that the accuracy of the incongruent trials was significantly lower than that of the congruent trials. The main effect of RSI was significant, *F*_(2,96)_ = 3.61, *p* = 0.031, ${\text{η}}_{\text{p}}^{2}$ = 0.07, due to the accuracy gradually increasing with RSI from short to long. The Congruency × RSI interaction was significant, *F*_(2,96)_ = 5.14, *p* = 0.008, ${\text{η}}_{\text{p}}^{2}$ = 0.10. The *post-hoc* tests indicated that the main effect of RSI was significant only in incongruent trials, *F*_(2,47)_ = 5.16, *p* = 0.009, ${\text{η}}_{\text{p}}^{2}$ = 0.18. The remaining main and interaction effects were not significant (*p*s > 0.08). Regarding the RTs, the main effect of Congruency was significant, *F*_(1,48)_ = 399.26, *p* < 0.001, ${\text{η}}_{\text{p}}^{2}$ = 0.89, which showed that the RTs observed during incongruent trials were significantly slower than those observed during congruent trials. The main effect of RSI was significant, *F*_(2,96)_ = 57.23, *p* < 0.001, ${\text{η}}_{\text{p}}^{2}$ = 0.54, and the RTs gradually decreased with RSI from short to long. The remaining main and interaction effects were not significant (*p* > 0.09).

**Table 2:** Accuracy and response times (RTs) of congruent and incongruent trials during different response stimulus intervals (RSIs) (M ± SD).

	**RSI (ms)**	**Congruent**		**Incongruent**	
		**Stress group**	**Control group**	**Stress group**	**Control group**
Accuracy (%)	200	94 ± 5	96 ± 3	92 ± 5	93.59 ± 3
	700	94 ± 6	96 ± 3	90 ± 7	91.87 ± 6
	1,200	93 ± 6	96 ± 3	92 ± 6	93.92 ± 4
RT(ms)	200	770 ± 44	776 ± 57	881 ± 70	900.69 ± 85
	700	729 ± 60	730 ± 58	848 ± 77	862.49 ± 74
	1,200	730 ± 67	726 ± 60	851 ± 84	842.58 ± 64

#### Errors and post-error responses

The Trial Type × Group ANOVA of the RTs observed during the correct and error trials showed that the main effect of Trial Type was significant, *F*_(1,48)_ = 38.28, *p* < 0.001, ${\text{η}}_{\text{p}}^{2}$ = 0.44, and the RTs observed during the error responses (846 ± 82 ms) were slower than those observed during the correct responses (803 ± 59 ms). On the other hand, the main effect of Group and the interaction effect were not significant (*p*s > 0.70).

**Figure 3:**
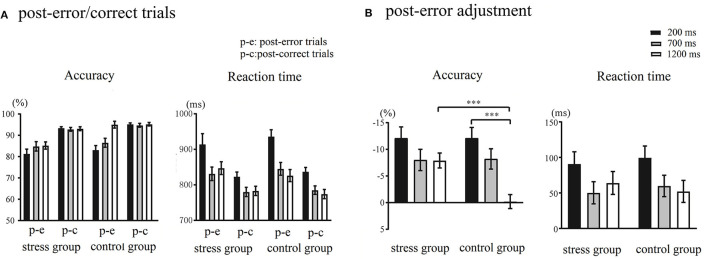
**(A)** Accuracy and response times (RTs) of post-error/correct trials during different response stimulus intervals (RSIs) in the control and stress groups. **(B)** The post-error adjustment in accuracy and RTs in the control and stress groups. The calculation formula of anterior cingulate cortex (ACC): ACC _post−error adjustment_ = ACC _post−error trial_ – ACC _post−correct trial_. The calculation formula of RTs: RTs _post−error adjustment_ = RTs _post−error trial_ – RTs _post−correct trial_. ***p < 0.001.

The accuracy and RTs of post-error/correct trials during different RSIs are shown in Figure 3A. Regarding accuracy, Trial Type × RSI × Group ANOVA showed that the main effect of Trial Type was significant, *F*_(1,48)_ = 86.32, *p* < 0.001, ${\text{η}}_{\text{p}}^{2}$ = 0.64, indicating that the accuracy of post-error trials was significantly lower than that of post-correct trials. The main effect of RSI was significant, *F*_(2,96)_ = 10.84, *p* < 0.001, ${\text{η}}_{\text{p}}^{2}$ = 0.18, with the longer the RSIs, the higher the accuracy. The main effect of Group was significant, *F*_(1,48)_ = 4.81, *p* = 0.03, ${\text{η}}_{\text{p}}^{2}$ = 0.09, showing that the accuracy of the stress group was significantly lower than that of the control group. The Trial Type × RSI × Group interaction was significant, *F*_(2,96)_ = 3.95, *p* = 0.02, ${\text{η}}_{\text{p}}^{2}$ = 0.08. The follow-up analysis indicated that under the 1,200 ms RSI, there was a significant difference in the accuracy between the post-error trials and post-correct trials in the stress group, *F*_(1,48)_ = 33.82, *p* < 0.001, ${\text{η}}_{\text{p}}^{2}$ = 0.41, but not significantly within the control group (*p* = 0.89). Furthermore, at 1,200 ms RSI, there was a significant difference in the accuracy of post-error trials between the stress group and control group, *F*_(1,48)_ = 17.81, *p* < 0.001, ${\text{η}}_{\text{p}}^{2}$ = 0.27, but there was no significant difference between post-correct trials (*p* = 0.09). Analysis of RTs revealed a main effect of Trial Type, *F*_(1,48)_ = 79.58, *p* < 0.001, ${\text{η}}_{\text{p}}^{2}$ = 0.62, as the RTs observed during post-error trials were significantly slower than those observed during post-correct trials. The main effect of RSI was significant, *F*_(2,96)_ = 56.74, *p* < 0.001, ${\text{η}}_{\text{p}}^{2}$ = 0.54, and the longer the RSIs, the faster the RTs. The Trial Type × RSI interaction was significant, *F*_(2,96)_ = 4.90, *p* = 0.009, ${\text{η}}_{\text{p}}^{2}$ = 0.09. The *post-hoc* tests revealed that the difference between the RTs observed during 700 and 1,200 ms RSIs was not significant. The main effect of Group and the remaining interaction effects were not significant (*p*s > 0.07). The post-error adjustment in accuracy and RTs during different RSIs are illustrated in Figure 3B. This study indicated the post-error adjustment by calculating the difference between the trials after the error responses and the correct responses under the same RSI.

## Discussion

This study used the Trier Social Stress Task and the color-word Stroop task to explore the processing of different stages in post-error adjustment following acute stress. Compared with the control group, the stress group showed increased salivary cortisol levels, heart rate, perceived stress levels, and negative affect, indicating that the induction of acute stress was successful. The results showed that the PES effect appeared in both the stress and control groups, and a trend for significantly greater PES at short RSIs, compared to long RSIs. The main effect of RSI on the accuracy of the post-error adjustment was significant only in the control group. The accuracy of the post-error adjustment increased over time, and the amount of the post-error adjustment was significantly higher at the longest RSIs in the control group but not in the stress group. Different from the control group, the accuracy of post-error trials at 1,200 ms RSI did not increase accordingly, which was close to the accuracy under 700 ms RSI and was significantly lower than that in the control group.

In this study, the PES effect appeared in the control group at all stages of post-error processing, and the accuracy of post-error trials was significantly lower than that of the post-correct trials. Moreover, with the prolongation of RSIs, the accuracy of post-error trials gradually increased and was close to that of the post-correct trials in the long RSI. These results indicated a poor task performance in the early stage of post-error processing, and adaptive regulation can only occur later. Contrary to the traditional accounts which propose that post-error slowing stems from top-down cognitive control or impaired task-related processing, this study suggested that the processing mechanism of post-error adjustment varied in multiple stages (Purcell and Kiani, 2016; Ullsperger and Danielmeier, 2016). Error monitoring includes cognitive processing such as error detection, error awareness, and error cause assessment (Ullsperger et al., 2014a,b). Error monitoring consumed attention resources, which reduced the cognitive processing resources of post-error trials to a certain extent, and it would cause interference in the early stage processing (Lavro and Berger, 2015; Van der Borght et al., 2016; Li et al., 2021). In this way, post-error responses' speed slowed down, but with no improvement in accuracy. More recently, Buzzell et al. (2017) found that the error positivity (Pe) amplitude shared a negative correlation with the P1 amplitude in the subsequent trials under short RSIs (200–533 ms). P1 component is one of the earliest event-related potential (ERP) components indicating early visual information processing (Luck et al., 1990; Di Russo et al., 2002). The above study disclosed that the cumulative evidence processing for error responses interferes with early perceptual processing in subsequent trials. Our result of the interference effect was consistent with this study.

In addition, with the extension of RSIs, the accuracy in post-error trials gradually increased, close to the post-correct trials in the longest RSI only in the control group. These results manifest adaptive regulation during the late stages of post-error processing in the participants without acute stress. Consistent with studies which have found that acute stress impairs cognitive control (e.g., Arnsten, 2009, 2015), the stress levels of catecholamines and cortisol lead to impaired PFC, including the lateral PFC, which regulates top-down selective attention processing (Banich, 2009; Katsuki and Constantinidis, 2012). It thus appears that acute stress impaired selective attention regulation processing. Adaptive post-error regulation is based on top-down selective attention, which could increase the modulation of task-related processing (King et al., 2010; Danielmeier et al., 2015; Purcell and Kiani, 2016). Actually, much evidence implicates post-error accuracy improvement occurs in post-error processing when the task-related selective attention focuses on the task-related motor or sensory processing (Maier et al., 2011; Van der Borght et al., 2016). The dysfunction of cognitive control under acute stress brought out individuals not effectively focusing their selective attention resources on tasks and even task dimensions related to error responses, making it difficult to adjust and promptly improve current task performance in the late processing stage of post-error adjustment. Notably, we did not observe a significant improvement in the accuracy after error responses (−0.18 ± 5.77%) under the 1,200 ms RSI condition in the control group. In fact, after an individual analysis of the post-error adjustment during the 1,200 ms RSI in the control group, we found that 65.38% of the participants had higher post-error accuracy than in the post-correct trials. Overall, there has been a trend toward a post-error accuracy improvement under the long RSI in this experiment, suggesting adaptive adjustment in the late stage of post-error processing.

Consistent with our previous study (Hu et al., 2019), this study did not reveal a stress effect on interference inhibition. There are many differences between post-error adjustment and interference inhibition, such as conflict/error monitoring, regulation strategies, and speed–accuracy trade-offs. However, the stress effect on inhibition remains elusive (Shields et al., 2016a). In addition, even though the lateral prefrontal cortex (LPFC) function was impaired following stress (Qin et al., 2009; Takizawa et al., 2014; Yennu et al., 2016), the familiarity with the Stroop task in individuals may compensate for the negative effect of acute stress on the interference inhibition processing after nearly 1,000 trials of practice. The stress effect on the Stroop task still needs further research.

In general, our research is consistent with the post-error multi-stage processing theory. A post-error adjustment involves different processing stages (Murphy et al., 2016; Steinhauser et al., 2017; Wessel, 2018), including both maladaptive and adaptive processing stages. Specifically, when attention resources are distributed to error monitoring, the processing of the subsequent trial would be distracted and limited in a relatively short period. At the late stage, the individual could improve performance through top-down cognitive regulation. For a long time, researchers have proposed that post-error processing was regulated by top-down cognitive control. Although post-error accuracy is an effective indicator of adaptive regulation after error responses, very few studies have revealed an improvement in post-error accuracy. Even studies that recruited patients with prefrontal lobe lesions have not reported related results (Ornstein et al., 2009; Wessel et al., 2014; Sullivan et al., 2019). The PIA may be vulnerable to specific experimental situations and experimental conditions. Our study has revealed the stage and features of post-error adaptive regulation through stress-induced impairment of top-down executive control without any physical trauma. This study provides new evidence for the phasic and adaptive features of post-error regulation.

Some limitations have to be mentioned in the present study. First, to exclude gender and menstrual cycle modulation of stress responses, we only recruited male participants in this study. Since several studies have revealed gender effects on cognitive processing (Shields et al., 2016b), further research could consider examining sex effects on post-error processing following acute stress. Second, some previous studies have shown no consistent conclusion about the post-error response following stress (Cavanagh and Allen, 2008; Hu et al., 2019), which might be affected by multiple factors such as experimental paradigm, error awareness, stress responses, gender, and individual personality characteristics. It still requires extensive research to explore.

## Conclusion

In summary, the present study showed stress-induced maladaptive adjustment only in the late stage of post-error processing, which manifests adaptive attention regulation in the late stage of post-error adjustment. This study provides new evidence for the post-error multi-stage processing theory.

## Data availability statement

The raw data supporting the conclusions of this article will be made available by the authors, without undue reservation.

## Ethics statement

The studies involving human participants were reviewed and approved by South-West University Human Ethics Committee for Human Research. The patients/participants provided their written informed consent to participate in this study.

## Author contributions

NH, QLi, and QLo contributed to conception and design of the study. NH and AC finished the manuscript. MD, MY, and XW contributed to the collection of research data. All authors contributed to the article and approved the submitted version.

## Funding

This work was supported by grants from the National Natural Science Foundation of China (32171040 and 32200878), the Annual 2021 Educational Science Planning Project of Yunnan Province (BFSJY006), and Kunming University Talent Introduction Research Project (YJW2213).

## Conflict of interest

The authors declare that the research was conducted in the absence of any commercial or financial relationships that could be construed as a potential conflict of interest.

## Publisher's note

All claims expressed in this article are solely those of the authors and do not necessarily represent those of their affiliated organizations, or those of the publisher, the editors and the reviewers. Any product that may be evaluated in this article, or claim that may be made by its manufacturer, is not guaranteed or endorsed by the publisher.
